# Research on the potential mechanisms of propofol in treating depression after brain injury based on network pharmacology

**DOI:** 10.1097/MD.0000000000046463

**Published:** 2025-12-12

**Authors:** Feng-Ting Ma, Bi-Hua Xie, Qiu-Xia Xiao, Liu-Lin Xiong, Fei Liu

**Affiliations:** a Department of Anesthesiology, The First People’s Hospital of Shuangliu District, West China (Airport) Hospital, Sichuan University, Chengdu, China; b Department of Anesthesiology, West China Hospital, Sichuan University, Chengdu, China; c Department of Anesthesiology, The Third People’s Hospital of Yibin, Yibin, Sichuan, China; d Department of Anesthesiology, The Third Affiliated Hospital of Zunyi Medical University (The First People’s Hospital of Zunyi), Zunyi, Guizhou, China.

**Keywords:** depression, ischemic brain injury, network pharmacology, propofol, traumatic brain injury

## Abstract

**Background:**

The precise mechanisms by which propofol exerts neuroprotective and antidepressant effects have not been fully elucidated. This study aimed to clarify how propofol alleviates depression following brain injury using a network pharmacology approach.

**Methods:**

By implementing strategies rooted in network pharmacology, we predicted shared target genes of propofol relevant to the treatment of traumatic brain injury, ischemic brain injury, and depression. We analyzed interactions and pathway enrichment through protein–protein interaction networks, along with Gene Ontology and Kyoto Encyclopedia of Genes and Genomes enrichment analyses. Potential protein targets were subjected to molecular docking with propofol using AutoDock Vina software.

**Results:**

After screening, we identified 22 intersection targets corresponding to 88 Gene Ontology entries and 20 Kyoto Encyclopedia of Genes and Genomes signaling pathways. The key targets primarily include core proteins such as gamma-aminobutyric acid receptors, glutamate receptors, dopamine receptors, and cation channels. Molecular docking simulations confirmed the stable binding of propofol to the 10 key targets.

**Conclusion:**

This study sheds light on the possible mechanisms by which propofol affects depression in the aftermath of brain injury. Future studies should utilize a network pharmacology framework to explore further the corresponding targets and pathways implicated in the therapeutic effect of propofol on depression after brain injury.

## 1. Introduction

Traumatic brain injury (TBI) and ischemic brain injury (IBI) represent 2 significant global health challenges, characterized by high incidence rates, disability, and mortality.^[1,2]^ TBI is caused by an external impact on the head, inducing damage to brain tissue, whereas IBI arises due to vascular obstruction, causing hypoxia and ischemia in specific brain areas. Despite distinct initial causes, both conditions can trigger secondary brain injuries, marked by similar pathological and physiological alterations. Secondary brain injury involves a cascade of cellular and metabolic changes, such as excitotoxicity, oxidative stress, mitochondrial disorders, inflammatory responses, and blood-brain barrier breakdown.^[3]^ Neurological dysfunction caused by secondary brain injury has always been a challenge and focus of clinical treatment and is also a major reason for high disability rates in patients. Depression frequently emerges as a common mental aftermath following brain injury, significantly affecting patient prognosis and quality of life and increasing mortality rates.^[4,5]^ The mechanisms underlying post-brain injury depression remain unclear but likely involve damage to brain functional networks, emotional regulation areas, neuroinflammation, and disruptions in the neurotransmitter system.

Propofol is a widely used short-acting intravenous anesthetic that exerts a sedative-hypnotic effect by activating the central inhibitory neurotransmitter gamma-aminobutyric acid (GABA). Early studies suggested that propofol protects the brain by reducing cerebral blood flow, intracranial pressure, and cerebral metabolic rate. As research progresses, it has become evident that the interaction between propofol and its receptors, along with a series of pathophysiological changes and alterations in gene expression induced by downstream signaling, may constitute the primary mechanism underlying its neuroprotective effects.^[6]^ In recent years, numerous scholars have discovered that, in addition to its neuroprotective properties, propofol also exhibits antidepressant effects. A clinical study demonstrated that high-dose propofol administration significantly alleviated symptoms of treatment-resistant depression in patients.^[7]^ Furthermore, a recent animal study indicated that propofol rapidly reverses anhedonia associated with chronic stress by directly inhibiting the dopamine transporter and activating D1-type medium spiny neurons (D1-MSNs) in the nucleus accumbens.^[8]^ Propofol is a typical single-agent, multi-target drug that exhibits multi-site, multi-target, and multi-mechanism properties. Consequently, we hypothesized that propofol may have therapeutic potential for treating depression following brain injury.

Network pharmacology emphasizes multi-target research strategies and investigates the pathological processes of diseases at the genetic level by constructing a disease–gene–target–drug interaction network.^[9]^ However, the molecular target mechanism of propofol in post-brain-injury depression remains unclear. Therefore, this study aimed to explore the potential mechanisms by which propofol may treat depression following TBI and IBI, using a network pharmacology approach. Additionally, it seeks to provide a theoretical foundation for further research on the effects of propofol on depression after brain injury and to support the clinical pharmacological understanding of this condition.

## 2. Materials and methods

### 2.1. Prediction of propofol targets

The 2D and 3D structural formulas of propofol were sourced from the PubChem database (https://pubchem.ncbi.nlm.nih.gov/).^[10]^ To ensure that we identified all potential target proteins for propofol, we searched databases for propofol, SwissTargetPrediction,^[11]^ SuperPred,^[12]^ DrugBank,^[13]^ and SEA.^[14]^ Our search was restricted to “*Homo sapiens*,” with a likelihood of exceeding 0. The UniProt protein database (http://www.uniprot.org/uploadlists/) was used to standardize all gene names, making them uniform and easy to use.

### 2.2. Prediction of targets related to TBI, IBI, and depression

We used multiple databases, GeneCards (http://www.Genecards.org),^[15]^ OMIM (http://omim.org),^[16]^ and DisGeNET (https://disgenet.com) databases^[17]^ to search for target genes related to TBI, IBI, and depression, with the search terms “craniocerebral injury” and “traumatic brain injury”; “ischemic brain injury”; and “depression,” respectively. After screening and deleting repetitive target genes, a collection of target genes for each of the 3 diseases was obtained. Then, all disease targets collected from the above databases were merged and the same genes were deleted to obtain a collection of target genes for each of these 3 diseases.

### 2.3. Construction of protein–protein interaction networks

We used the Venny 2.1.0 tool (available at https://bioinfogp.cnb.csic.es/tools/venny/) to develop a visual crossover diagram illustrating the interaction between isoproterenol and 3 distinct diseases. Subsequently, the identified target gene intersections were submitted to the STRING database (https://string-db.org/). In this platform, we concentrated on “multiple proteins,” refined our query to “*Homo sapiens*,” and established an interaction score threshold at a medium confidence level of 0.400. Additionally, we excluded any isolated proteins to enhance the clarity of our analysis, leading to a well-organized protein–protein interaction (PPI) network. We further optimized and examined the PPI network using CytoScape version 3.10.0. The maximum connectivity cluster was determined using the cytoHubba tool to filter the top 10 targets identified as hub targets.

### 2.4. Enrichment analysis of Gene Ontology and Kyoto Encyclopedia of Genes and Genomes

The overlapping targets were subjected to functional enrichment analysis for Gene Ontology (GO) and Kyoto Encyclopedia of Genes and Genomes (KEGG) pathways using the DAVID database (https://davidbioinformatics.nih.gov/).^[18]^ The species selected for this analysis was “*Homo sapiens*,” with OFFICIAL_GENE_SYMBOL as the type, a *P*-value threshold of .05, and the resulting enrichment data were filtered and downloaded. Weishengxin (http://www.bioinformatics.com.cn/) serves as an online platform for data mapping and analysis, utilizing Python along with various R packages.^[19]^ Using the Weishengxin platform, the top 10 entries with the most significant *P*-values of GO function enrichment were selected for graphical visualization. The KEGG pathways that exhibited *P*-values <.05 were chosen for representation in a bubble plot forma, and the pathways involved in the 10 key targets were displayed as a Chordal graph.

### 2.5. Construction of propofol-target-pathway-disease network

We gathered detailed information regarding the drug, intersecting targets, pathways, and diseases, and created a “drug-target-pathway-disease” network utilizing CytoScape 3.10.0 software to illustrate propofol’s interactions with the 3 diseases.

### 2.6. Molecular docking

Molecular docking techniques were utilized to model the interaction between propofol molecules and their targets, forecasting both the mode of binding and the capacity for binding. The crystal structures of the 10 most significant targets were sourced from the RCSB Protein Data Bank (http://www.rcsb.org/), with “*Homo sapiens*” designated as the species, “X-RAY DIFFRACTION” identified as the experimental methodology, and “Protein” as the type of polymer entity. A protein crystal with lower resolution was selected as the docking template. Using AutoDock Tool,^[20]^ water molecules were excluded, proteins were disentangled, nonpolar hydrogen atoms were introduced, and the Gasteiger charge of the structure was computed. These results were documented as receptors in the PDBQT format. The SDF file corresponding to the 3D structure of propofol was transformed to PDB format using Open Babel and later saved as docking ligands within AutoDock Vina software in PDBQT format. Subsequently, molecular docking involving the top 10 key targets with propofol was performed using AutoDock Vina to validate the interaction. The outcomes were depicted using the online platforms PLIP (https://plip-tool.biotec.tu-dresden.de/plip-web/plip/index)^[21]^ and PyMOL 2.5 software, which showcased the 3D configurations, protein residues, and binding interactions between proteins and small molecules.

## 3. Results

### 3.1. Propofol-related targets

**Figure 1.**
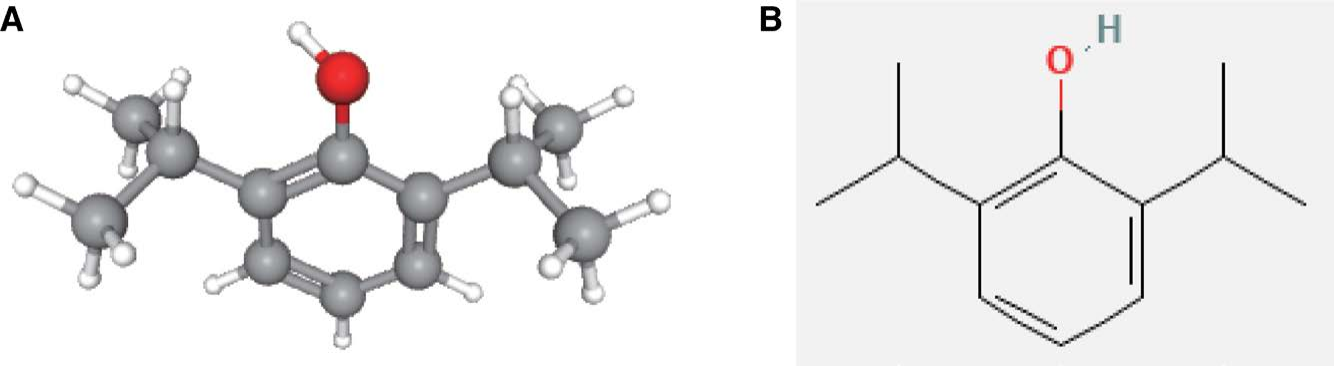
(A) 3D structure diagram of the propofol molecule. (B) Chemical structure of the propofol molecule. The 3D structure and chemical formula in this image are sourced from the PubChem database. (Images from PubChem may be downloaded and used without permission.)

The structures of propofol, including its chemical and 3D configurations (propofol|C12H18O|CID 4943 – PubChem, which was cited by https://pubchem.ncbi.nlm.nih.gov/compound/4943#section=2D-Structure), are illustrated in Figure 1. The search for targets associated with propofol utilized the SwissTarget Prediction, SuperPred, DrugBank, and SEA databases, yielding counts of 16, 79, 4, and 13, respectively. After eliminating duplicate entries, the overall propofol-related target count was 99.

### 3.2. Diseases-related targets (TBI, IBI, depression)

The related target genes of the 3 diseases were screened using the GeneCards database, and genes with a relevance score of ≥10 were selected. Subsequently, the screening results from the OMIM and DisGeNET databases were combined, duplicates were removed, and 1311 target genes for TBI, 1721 target genes for IBI, and 2019 target genes for depression were identified.

### 3.3. Intersection target PPI network

**Figure 2.**
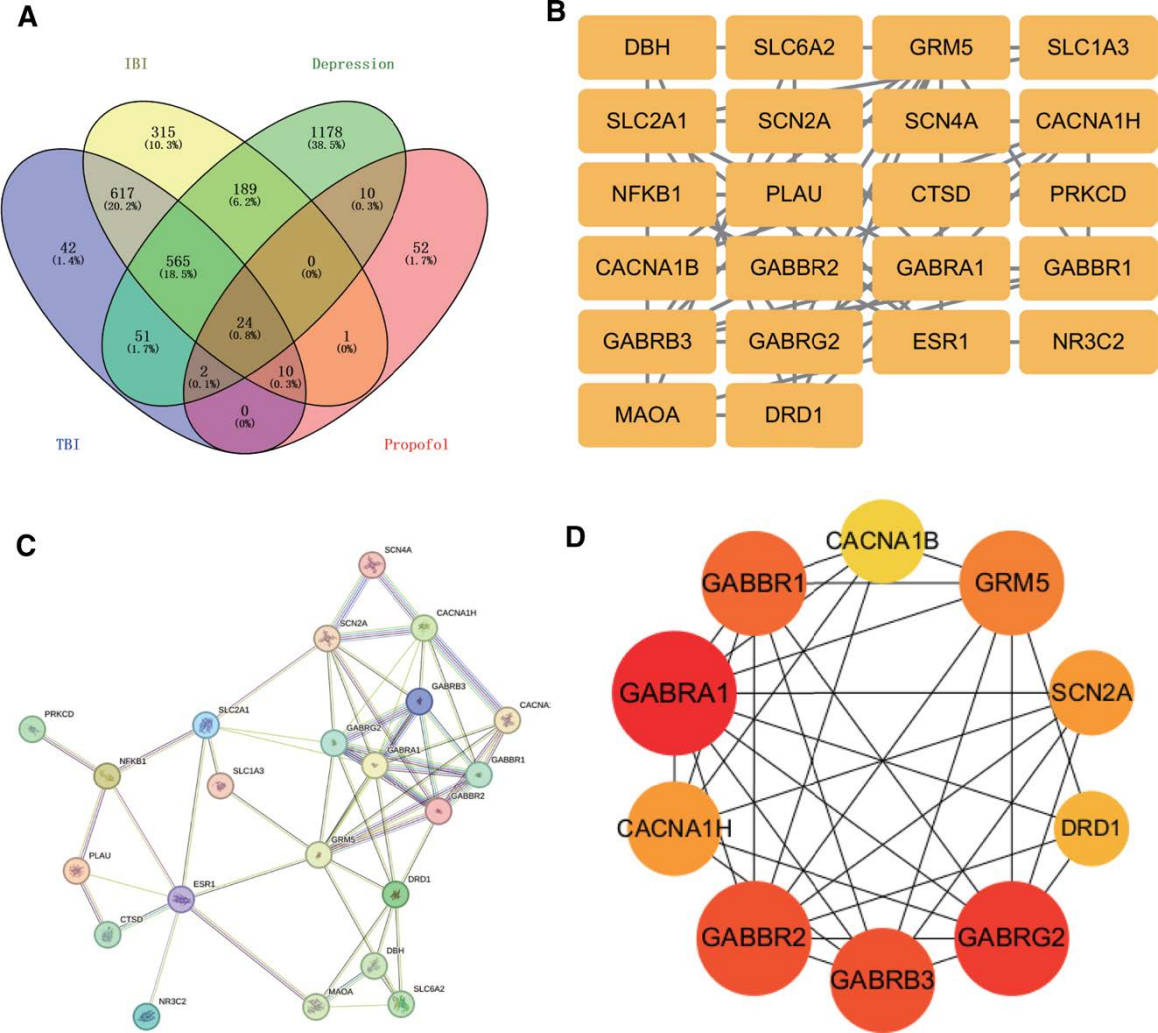
(A) Propofol targets and a Venn diagram of 3 diseases, and (B) 22 common intersection targets of propofol and 3 diseases. (C) PPI network diagram of intersection targets (STRING version). (D) PPI network diagram of key targets. PPI = protein–protein interaction.

The targets linked to propofol were analyzed in relation to the 3 diseases, with their intersections illustrated in a Venn diagram generated through the Venny 2.1 online tool, as shown in Figure 2A, resulting in 24 intersectional targets. The intersecting targets were submitted to the STRING database for PPI analysis. The isolated targets were removed, 22 targets were obtained, as depicted in Figure 2B, and the PPI network diagram was exported in Figure 2C. The PPI network was optimized and analyzed using Cytoscape 3.10.0 software. The CytoHubba tool was used to calculate maximum connectivity cluster ranking, and the top 10 targets were selected as key targets for constructing the PPI network diagram of key targets, which is plotted in Figure 2D. The significance of a target in the network is directly proportional to the intensity of its red color. The key targets selected were *GABRA1*, *GABRG2*, *GABBR2*, *GABRB3*, *GABBR1*, *GRM5*, *SCN2A*, *CACNA1H*, *DRD1*, and *CACNA1B*.

### 3.4. Enrichment analysis

**Figure 3.**
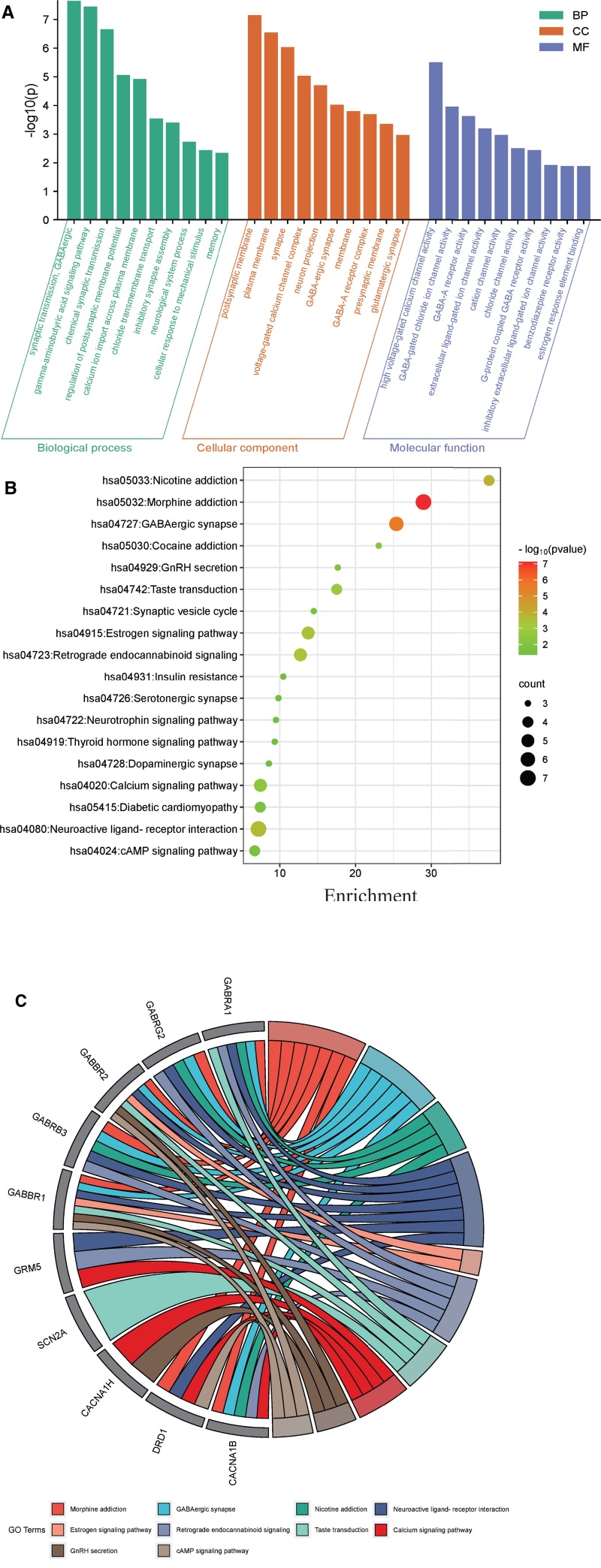
GO and KEGG enrichment analysis results. (A) GO function analysis histogram. BP is marked by dark cyan, CC is marked by sienna, and MF is marked by steel blue. (B) Dot plot of the KEGG pathway enrichment analysis. The horizontal axis represents the gene ratio, while the vertical axis represents the enriched pathway name. The color scale indicates different thresholds of the *P*-value, and the size of the dot indicates the number of genes corresponding to each pathway. (C) Chord diagram of KEGG pathway involving 10 key targets. BP = biological processes, CC = cellular components, GO = Gene Ontology, KEGG = Kyoto Encyclopedia of Genes and Genomes, MF = molecular functions.

PPI analysis led to the identification of various targets, which were then subjected to GO functional and KEGG pathway enrichment analyses. In total, 88 GO enrichment outcomes were recorded, comprising 38 related to biological processes (BP), 27 pertaining to cellular components, and 23 focusing on molecular functions (MF). Notably, the BP enriched in the GO analysis primarily included GABAergic synaptic transmission, GABA signaling pathway, chemical synaptic transmission, regulation of postsynaptic membrane potential, calcium and chloride transmembrane transport, cellular responses to mechanical stimuli, and neurological processes associated with sensory, motor, and cognitive memory. Cellular components were enriched mainly in the presynaptic and postsynaptic membranes, GABAergic synapses, glutamatergic synapses, GABA_A_ receptor complexes, voltage-gated calcium channel complexes, neuronal synapses, and cell membranes. The MF are mainly related to high voltage-gated calcium channels, GABA-gated chloride channels, GABA_A_ receptor activity, cation channel activity (e.g., sodium, potassium, calcium), G-protein–coupled GABA receptor activity, inhibitory extracellular ligand-gated ion channel activity, and the binding of estrogen-responsive elements. The enrichment outcomes were sorted by increasing *P*-values, and the top 10 entries were selected for graphical visualization, as shown in Figure 3A. In total, 20 KEGG pathways were enriched, with the primary pathways encompassing drug addiction, neurotransmission, hormone regulation, metabolic diseases, and the sensory system. The results of the pathways with *P*-value <.05 were visualized as a bubble plot in Figure 3B, where bubble color intensity indicates pathway importance and size reflects the number of enriched genes. Additionally, Figure 3C presents a chord diagram depicting the enriched KEGG pathways associated with the 10 key targets. This visualization offers a detailed and comprehensive representation of the intricate interactions and relationships among these pathways.

### 3.5. Propofol-target-pathway-disease network

**Figure 4.**
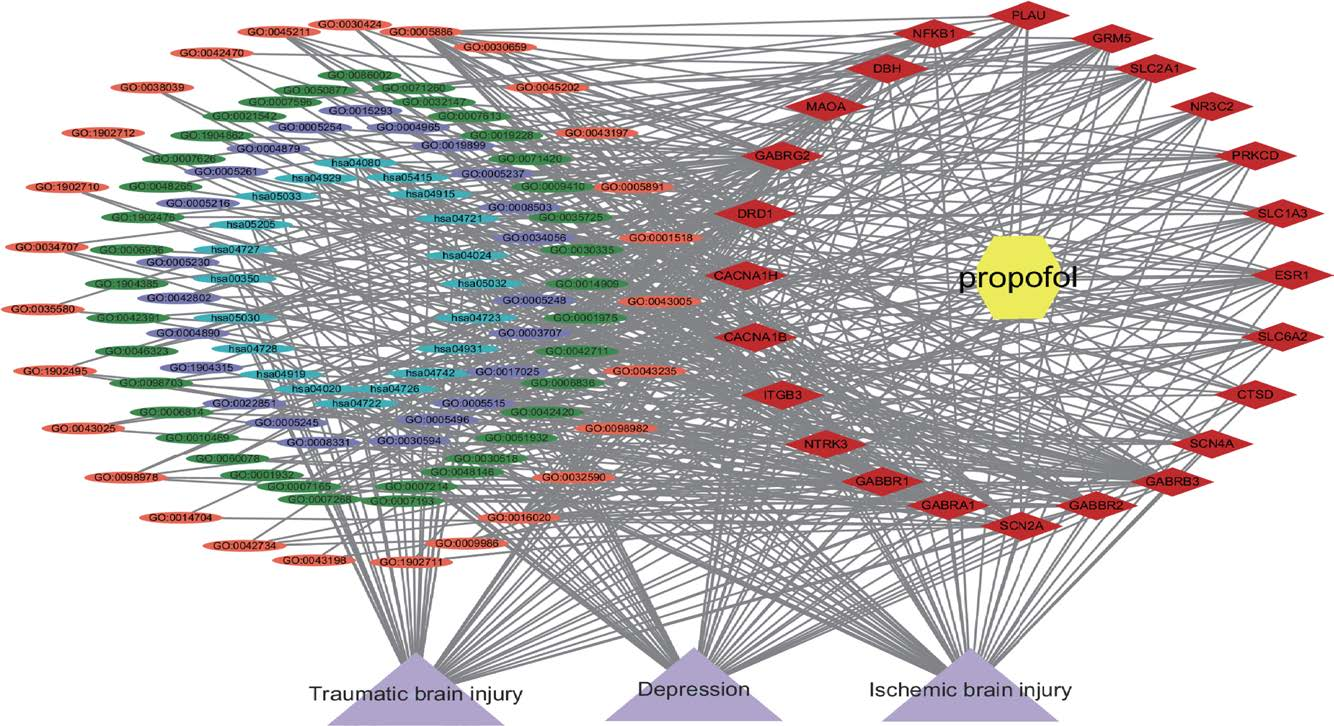
Drug-target-pathway network of propofol in the treatment of traumatic brain injury, ischemic brain injury, and depression (created using CytoScape 3.10.0 Software Mapping).

Utilizing propofol as a basis, along with intersecting target genes, GO enrichment for BP, and KEGG enrichment pathways related to diseases, CytoScape 3.10.0 software was employed to generate a drug-target-pathway network, as illustrated in Figure 4. The network consists of 136 nodes and 531 edges. Purple triangles represent diseases, yellow hexagons represent drugs, red rhombuses represent key targets, ellipses represent enrichment results, blue ellipses represent KEGG pathways, green ellipses represent BP, orange ellipses represent cellular components, and purple ellipses represent MF.

### 3.6. Analysis of molecular docking results

**Figure 5.**
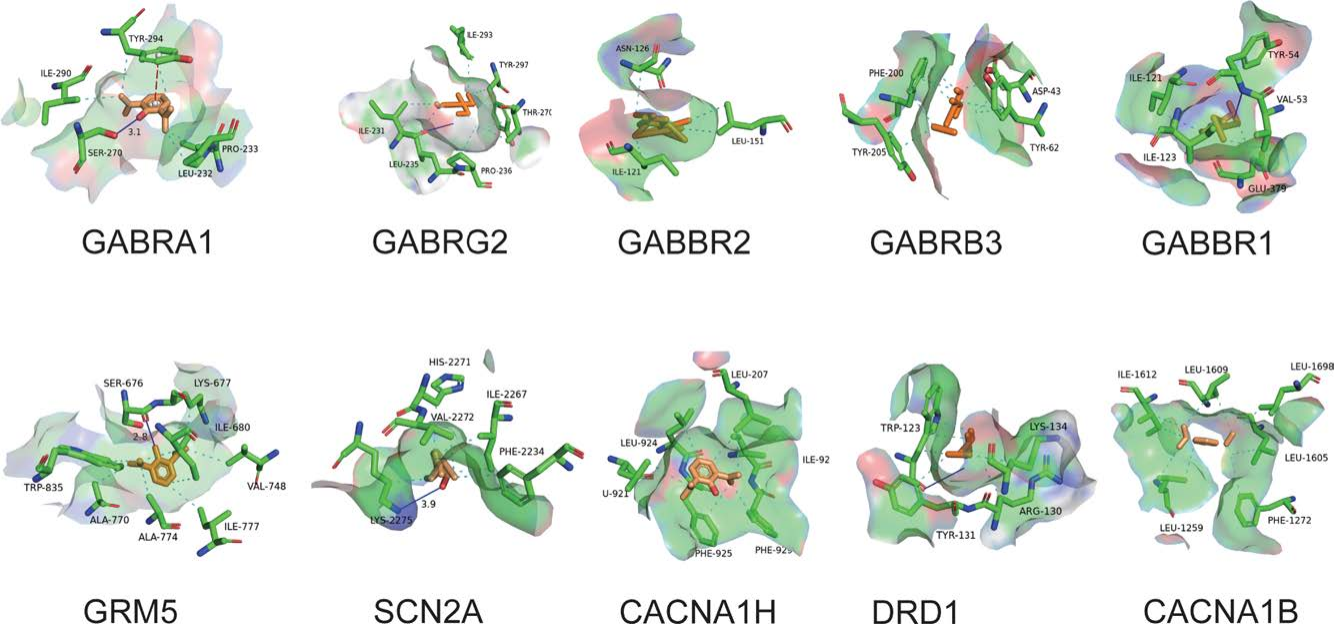
Molecular docking diagram of propofol and key targets (visualisation using the online tools PLIP and PyMOL2.5 software). The dark blue solid line represents hydrogen bonds, the light blue dashed line represents hydrophobic interactions, and the red dashed line represents π-π stacking.

In molecular docking, binding energy serves as an essential metric for evaluating the stability of the complex formed between a ligand and its corresponding target. A lower binding energy indicates a more stable complex, with a benchmark of −5 kcal/mol, generally implying strong binding affinity. The validation outcomes from docking propofol with 10 key target molecules in this study indicated that propofol demonstrated effective binding capabilities to the docked targets (Table 1). The results were visualized using the online tools PLIP and PyMOL2.5 software, as show in Figure 5. As shown in Figure 5, propofol and the target are bound to each other in a variety of ways, including hydrogen bonding, hydrophobic interactions, and intermolecular forces such as π–π stacking, resulting in a more stable conformation.

**Table 1:** Binding energies of propofol and key targets.

Target	PDB ID	The binding energy/Kal·mol^−1^
GABRA1	6D1S	−7.3
GABRG2	8BHG	−7.4
GABBR2	4F11	−6.9
GABRB3	4COF	−8.5
GABBR1	4MS4	−6.5
GRM5	6FFI	−6.8
SCN2A	4RLY	−6.3
CACNA1H	9AYG	−6.8
DRD1	7JOZ	−6.6
CACNA1B	7VFS	−7.9

## 4. Discussion

The extremely complex pathophysiological changes and unclear pathogenesis of post-brain injury depression present significant challenges for its treatment. Research conducted by Siddiqi et al found a decrease in the connection between the dorsal frontoparietal network and the subanterior cingulate cortex in patients with depression following TBI, while the connection between the dorsal frontoparietal network and the default mode network increased.^[22]^ They proposed that post-brain injury depression may represent a distinct clinical disorder independent of TBI, major depressive disorder, and post-traumatic stress disorder.^[22]^ Furthermore, many scholars acknowledge the clinical differences between post-brain injury depression and primary major depression, including poor response to traditional antidepressant medications and psychotherapy in post-TBI depression.^[5,23,24]^ Additionally, there is limited evidence supporting the efficacy of pharmacological or psychological treatments in reducing the prevalence of poststroke depression.^[25]^ Currently, there is a notable lack of targeted clinical interventions and evidence-based treatment guidelines for post-brain-injury depression.^[26]^

As research has progressed, investigations into propofol have broadened from merely its anesthetic properties to encompass its effects on neuroprotection, immune regulation, and potential as an antidepressant, which has garnered increasing attention in the realm of global scientific research. Utilizing a network pharmacology framework, this study identified potential targets of propofol that may be relevant for treating depression following TBI and IBI. The targets included *GABRA1*, *GABRG2*, *GABRB3*, *GABBR2*, *GABBR1*, *GRM5*, *DRD1*, *CACNA1H*, *CACNA1B*, and *SCN2A*. The neuroprotective and antidepressant effects of propofol may be related to the stimulation of GABA receptors, metabotropic glutamate receptor 5 (mGluR5), and dopamine D1 receptor (D1R), along with the modulation of sodium, calcium, and various other cation channels. These proteins predominantly participate in mechanisms associated with morphine addiction, GABAergic synapses, interactions between neuroactive ligands and receptors, estrogen signaling pathways, and retrograde signaling pathways involving endocannabinoids.

### 4.1. Propofol may exert its therapeutic effect on post-brain injury depression by acting on GABA receptors

GABA serves as the primary inhibitory neurotransmitter in the central nervous system and its receptors are categorized into 2 types: GABA_A_ and GABA_B_. GABA_A_ receptors belong to the ligand-gated chloride ion channel category, which regulate chloride ion channel opening, leading to membrane hyperpolarization and inhibition of neuronal excitability.^[27]^ Studies have demonstrated that propofol enhances the function of both presynaptic and postsynaptic GABA_A_ receptors, promoting the influx of chloride ions and thereby protecting neurons from mechanical damage. Additionally, propofol reduces tissue damage resulting from cerebral ischemia through the activation of GABAA receptors.^.[28]^ Furthermore, McMurray et al found that drugs acting on GABA_A_ receptors have antidepressant effects^[29]^ with pharmacological mechanisms related to the activation of the GABAergic system. GABAergic neuronal function and activity are significantly reduced in patients with treatment-resistant depression. Propofol, a GABA_A_ receptor agonist, can alleviate the learning and memory disturbances induced by electroconvulsive therapy.^[30]^

GABA_B_ receptors are important members of the G-protein–coupled receptor(GPCR) family. Upon ligand binding, they activate specific heterotrimeric G proteins that regulate intracellular calcium influx and potassium efflux, thereby inhibiting neuronal excitability.^[31]^ Research suggests that the recovery of GABA_B_ receptor expression after brain ischemia helps reduce neuronal excitability and mitigates the pathological damage caused by ischemia, thereby protecting neurons.^[32]^ Moreover, GABA_B_ receptors are involved in regulating slow, long-lasting neural responses and play a crucial role in cognitive functions, such as learning, memory, and synaptic signal transmission.^[33]^ Studies indicate that decreased expression levels of cell membrane GABAB receptors in the hippocampal and ventral tegmental area are associated with depression, whereas GABA_B_ receptor agonists can prevent depression development by enhancing the expression of these receptors.^[34]^ Given the potential role of GABA_B_ receptors in various neurological and psychiatric disorders, they are potential therapeutic targets for addiction, depression, and other conditions.^[35]^ The findings of this study suggest that propofol may stabilize GABA receptors, potentially regulating neurotransmitter release and synaptic plasticity, and exert multiple effects, such as anti-inflammatory, antioxidant, anxiolytic, and antidepressant actions.

### 4.2. GRM5 may be a new target for propofol in treating depression following brain injury

Metabotropic glutamate receptor 5 (mGluR5), encoded by GRM5, is a member of the GPCR family and is primarily distributed in key regions of the brain such as the cortex, hippocampus, striatum, and basal ganglia.^[36]^ mGluR5 in the postsynaptic membrane triggers intracellular calcium release and protein kinase C activation via signaling pathways involving phospholipase C and mitogen-activated protein kinase.^[37]^ In cases of acute brain injury or ischemia, protein levels of mGluR5 are upregulated, and increased expression is observed in neurons, astrocytes, and microglia in the cortex.^[38]^ Glutamate activates mGluR5, initiating a cascade of signal transduction pathways that mediates neuronal injury and death. mGluR5 agonists can prevent GluA2 downregulation caused by cerebral ischemia via the PI3K/Akt pathway and promote neuroprotective responses in astrocytes and microglia.^[39]^ Following TBI, the use of mGluR5 agonists significantly reduces brain edema and neuronal degeneration, with its activation potentially alleviating secondary brain damage post-TBI by modulating microglia-related neuroinflammation.^[40]^ Byrnes et al further indicated that in a mouse model of TBI, a single dose of mGluR5 agonist treatment reduced the number of activated microglia 1 month later, attenuated neuronal loss in the hippocampus, and notably enhanced the recovery of motor and cognitive functions, possibly through the modulation of neuroinflammation.^[41]^ Neuroinflammation is a key mechanism underlying depression following brain injury, where microglia and astrocytes play crucial roles and are closely associated with the onset of depression.^[42,43]^ Studies suggest that the expression of mGluR5 in astrocytes in the hippocampus can reverse behavioral and functional deficits induced by chronic stress.^[44]^ Restoration or enhancement of mGluR5 signaling in the ventral tegmental area may improve depression-like behaviors and pain sensitivity in mouse models of chronic social defeat stress via the endocannabinoid signaling pathway.^[45]^ mGluR5 serves as a target for neuroprotective drugs and has immense potential for the treatment of acute and chronic neurodegenerative diseases.^[46]^ Despite the lack of studies on the neuroprotective or antidepressant effects of propofol mediated by mGluR5, our findings suggest that this molecule could serve as a new target for the treatment of post-brain injury depression using propofol, providing new insights for basic research and clinical treatment.

### 4.3. Propofol’s potential mechanisms of antidepressant effects via D1R

D1R, encoded by the DRD1 gene, belongs to the class A GPCRs. It is widely found in areas such as the striatum, frontal cortex, substantia nigra pars reticulata, and adrenal medulla cells and plays a crucial role in synthesizing the intracellular second messenger cyclic adenosine monophosphate.^[47]^ Owing to its critical roles in cognitive and motor processes, D1R has emerged as a potential therapeutic target for various psychiatric disorders.^[48]^ Animal studies have shown that the activation of D1R neurons in the prefrontal cortex can rapidly produce anxiolytic and antidepressant effects.^[49]^ Excessive activation of the NLRP3 inflammasome in the cortex following brain injury leads to secondary neuroinflammation. Propofol can mitigate inflammatory responses and alleviate secondary brain damage by suppressing the activation of NLRP3 inflammasomes and facilitating the maturation of pro-inflammatory cytokines via the D1R pathway.^[50,51]^ Additionally, research by Hu Ji et al further elucidated the potential mechanisms through which propofol exerts its antidepressant effects via D1R. This study indicates that propofol directly binds to the dopamine transporter to block its transport function, thereby regulating dopamine concentrations in neuronal synapses.^[8]^ Furthermore, propofol participates in regulating depression-related anhedonic-like behaviors by promoting the activity of neurons expressing D1R in the striatum (NAC D1-MSNs).^[8]^ In a mouse model of chronic stress induction, propofol reversed long-term stress-induced anhedonic behavior, which was also attributed to the activation of D1-MSNs.^[8]^

### 4.4. Cation channels

Calcium and sodium ion channel proteins encoded by CACNA1H, CACNA1B, and SCN2A play crucial roles in regulating neuronal electrical activity and excitability. These channel proteins control the flow of cations, which are essential for maintaining the normal function of the nervous system. Propofol can block sodium ion channels, which helps reduce the release of the excitatory neurotransmitter glutamate, thereby lowering neuronal overexcitation and potential nerve damage.^[52]^ In addition to its blocking effect on sodium ion channels, propofol exerts neuroprotective effects by inhibiting intracellular calcium overload, which can prevent neuronal damage caused by excessive calcium ions. Propofol directly interacts with calcium channel proteins to block the influx of extracellular calcium ions.^[52]^ Furthermore, propofol can inhibit the activity of phospholipase C, thereby suppressing the synthesis of inositol triphosphate, which helps reduce the release of stored calcium ions inside cells, further alleviating intracellular calcium overload.^[53]^ Li et al further confirmed the neuroprotective effects of propofol on hippocampal neurons. They found that propofol effectively alleviates intracellular calcium overload caused by hypoxia-reoxygenation injury by inhibiting the activity of calcineurin and blocking the dissociation of FKBP12.6 from RyR.^[54]^

## 5. Conclusions

In conclusion, this study pioneered the investigation of potential targets and mechanisms by which propofol may aid in treating depression after brain injury. This lays a foundational theoretical framework for subsequent inquiries into the clinical pharmacological effects of propofol on depression resulting from brain injury. Moreover, this study introduces novel concepts for the clinical management and prevention of post-brain injury depression. However, a major limitation of this study is that it has not been confirmed using cellular or animal models. Further external experiments are necessary to substantiate the findings of future research.

## Author contributions

**Conceptualization:** Feng-Ting Ma, Bi-Hua Xie.

**Data curation:** Feng-Ting Ma.

**Formal analysis:** Feng-Ting Ma.

**Funding acquisition:** Fei Liu.

**Investigation:** Qiu-Xia Xiao.

**Methodology:** Feng-Ting Ma, Bi-Hua Xie.

**Project administration:** Liu-Lin Xiong.

**Resources:** Qiu-Xia Xiao.

**Software:** Feng-Ting Ma, Bi-Hua Xie.

**Supervision:** Liu-Lin Xiong, Fei Liu.

**Validation:** Qiu-Xia Xiao.

**Writing – original draft:** Feng-Ting Ma.

**Writing – review & editing:** Liu-Lin Xiong, Fei Liu.
